# Supplementary material for: Estimating COVID-19 Hospitalizations in the United States With Surveillance Data Using a Bayesian Hierarchical Model: Modeling Study
Source: JMIR Public Health Surveill. 2022 Jun 2;8(6):e34296. doi: 10.2196/34296 (PMC9169704; doi:10.2196/34296)
Supplement: Multimedia Appendix 1 [file publichealth_v8i6e34296_app1.docx]

## Multimedia Appendix 1

Table S1: Covariates selected for each Bayesian model for extrapolation of COVID-19 hospitalizations for all 50 U.S. states by age group out of all covariates.

| **0-17 years** | **18-49 years** | **50-64 years** | **65-74 years** | **75-84 years** | **85+ years** |
| --- | --- | --- | --- | --- | --- |
| %Positive   - %COVID inpat.   %ICU in use  %Minority | - %Positive - %COVID deaths - %COVID inpat. - %ICU in use - %Minority - %CKD - %COPD - %Diabetes - %Asthma | - %Positive - %COVID deaths - %COVID inpat. - %ICU in us - %Minority - %Heart Disease - %Obesity - %CKD - %Diabetes | - %Positive - %COVID deaths - %COVID inpat. - %ICU in use - %Minority - %Obesity - %COPD - %Diabetes | - %Positive - %COVID deaths - %COVID inpat. - %ICU in use - %CKD - %Heart Disease - %Obesity - %COPD - %Diabetes | - %Positive - %COVID deaths - %COVID inpat. - %ICU in use - %Obesity - %COPD - %Diabetes |

Footnote: ICU=intensive care unit, inpat. = inpatients, COPD= chronic obstructive pulmonary disease, CKD=chronic kidney disease; For the 0-17 years age group, only asthma was included as a possible covariate from the chronic conditions/diseases.
