# Supplementary figures and images for: Estimating COVID-19 Hospitalizations in the United States With Surveillance Data Using a Bayesian Hierarchical Model: Modeling Study
Source: JMIR Public Health Surveill. 2022 Jun 2;8(6):e34296. doi: 10.2196/34296 (PMC9169704; doi:10.2196/34296)

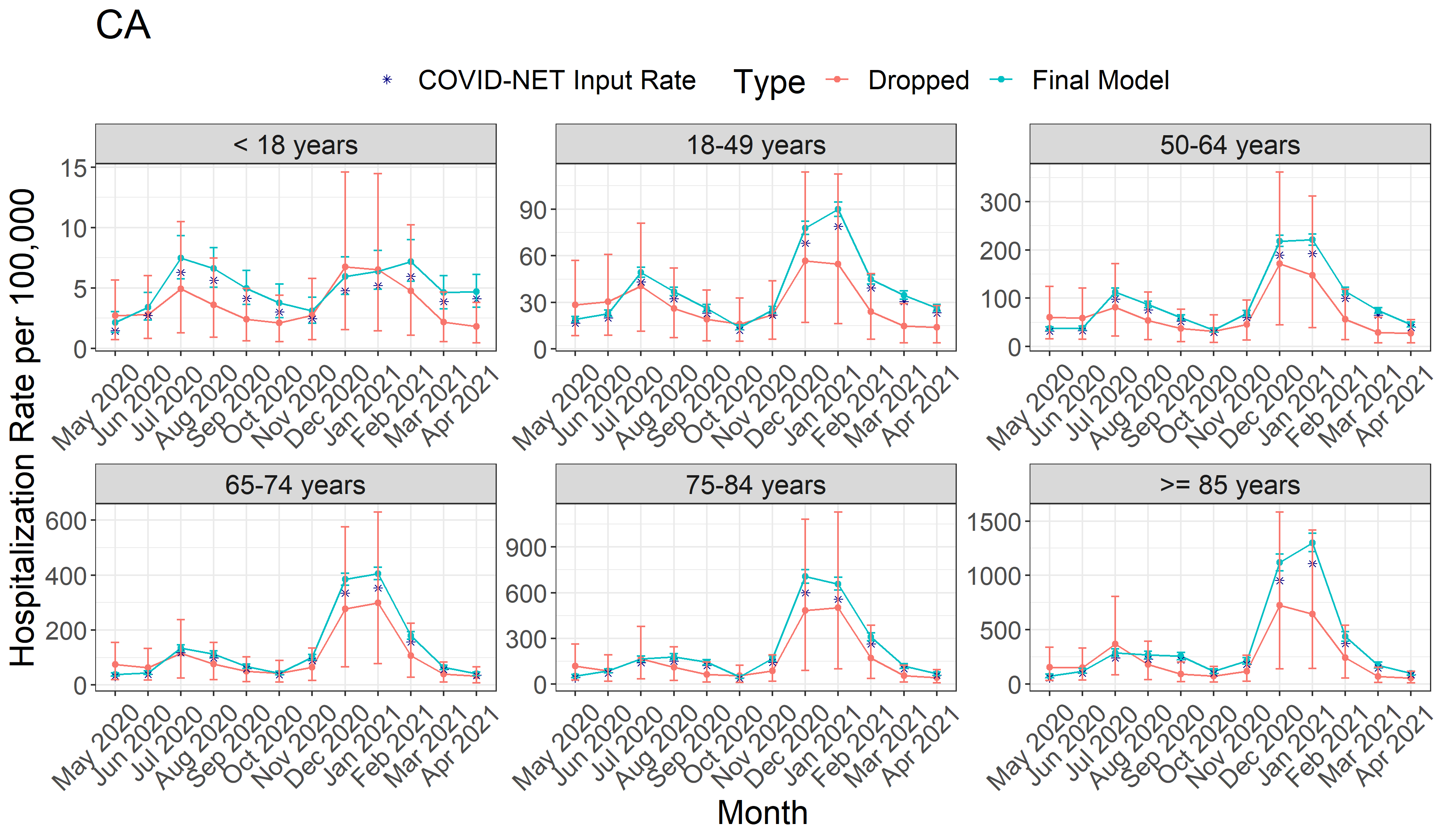


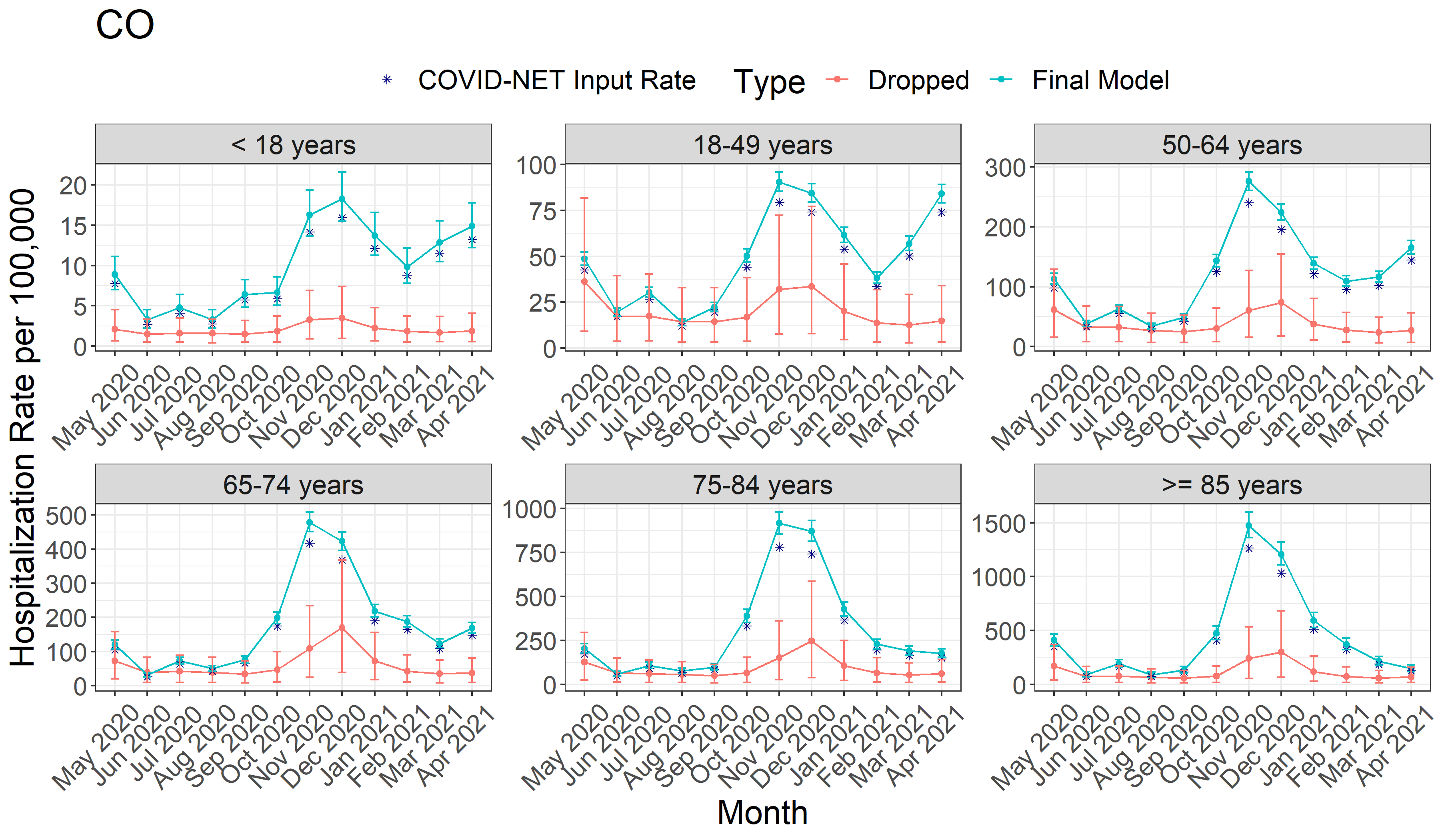


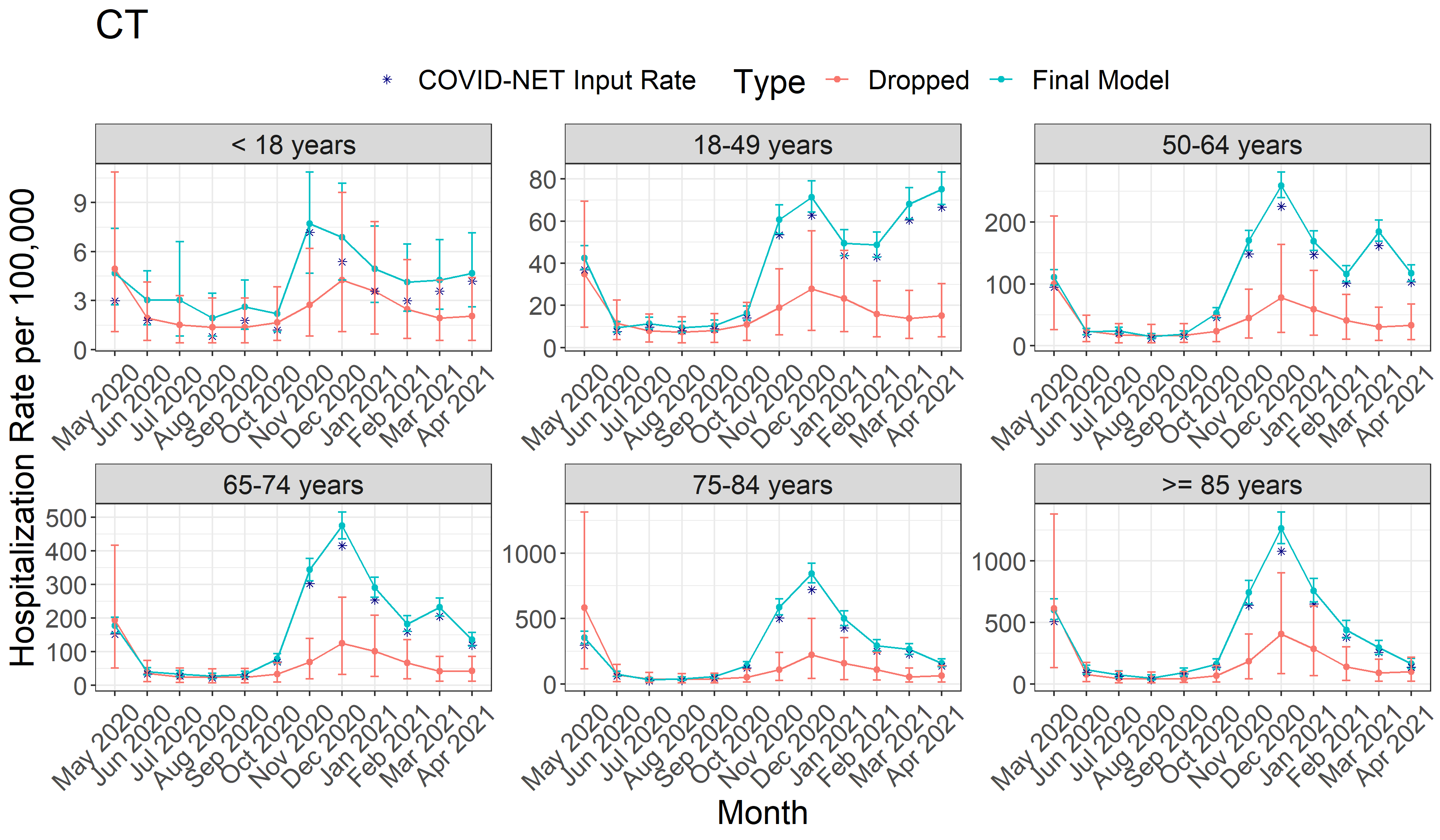


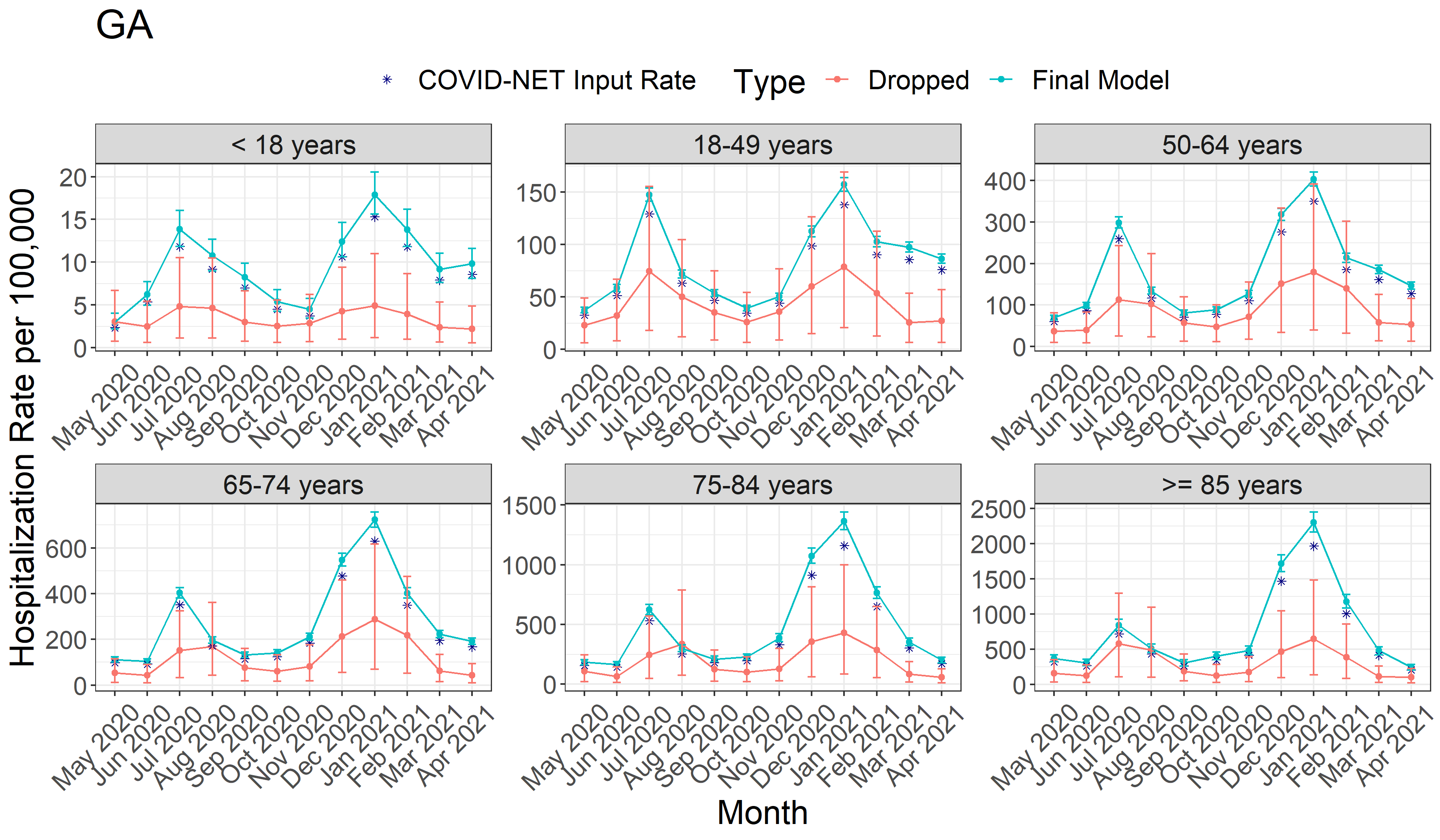


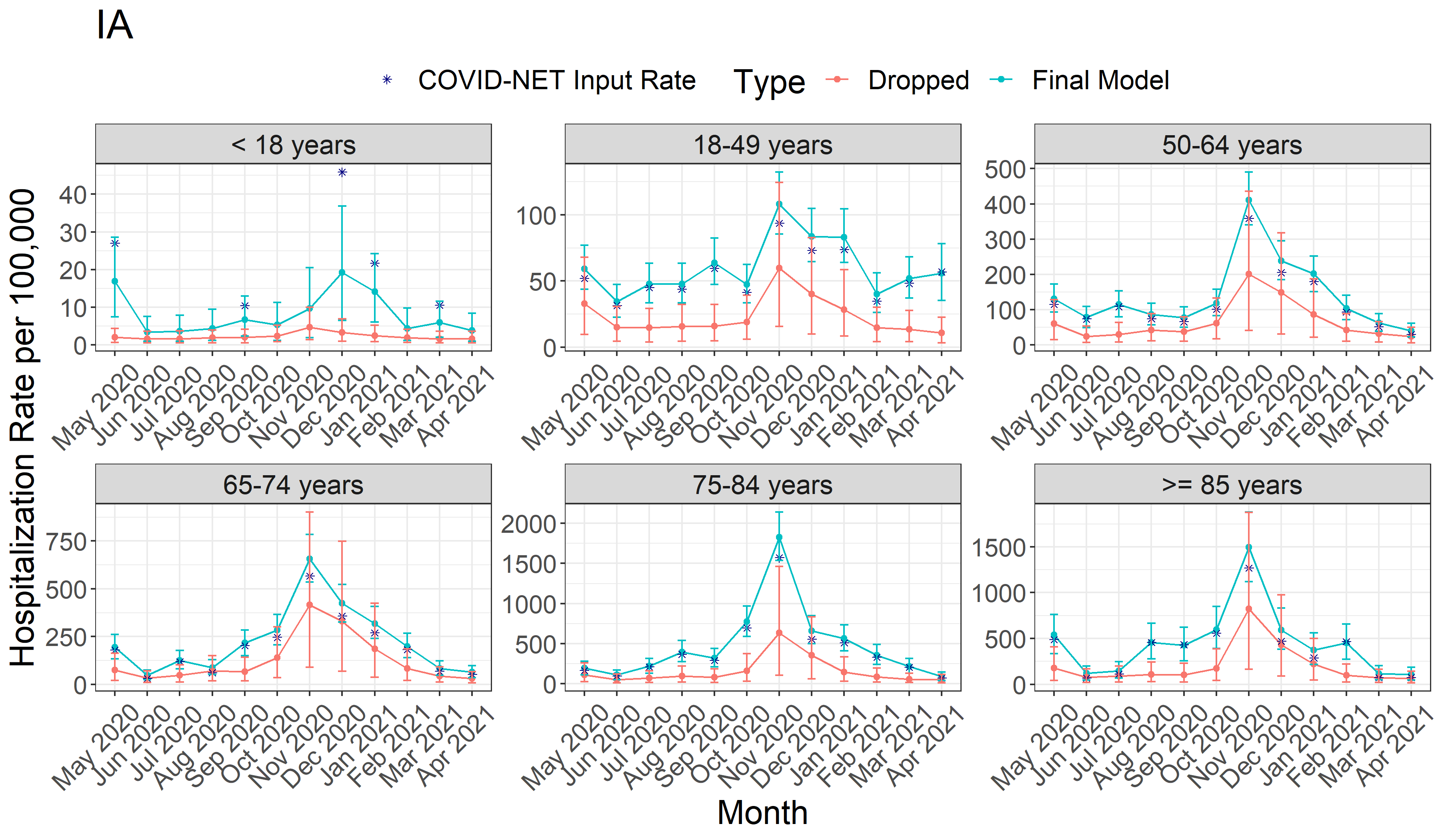


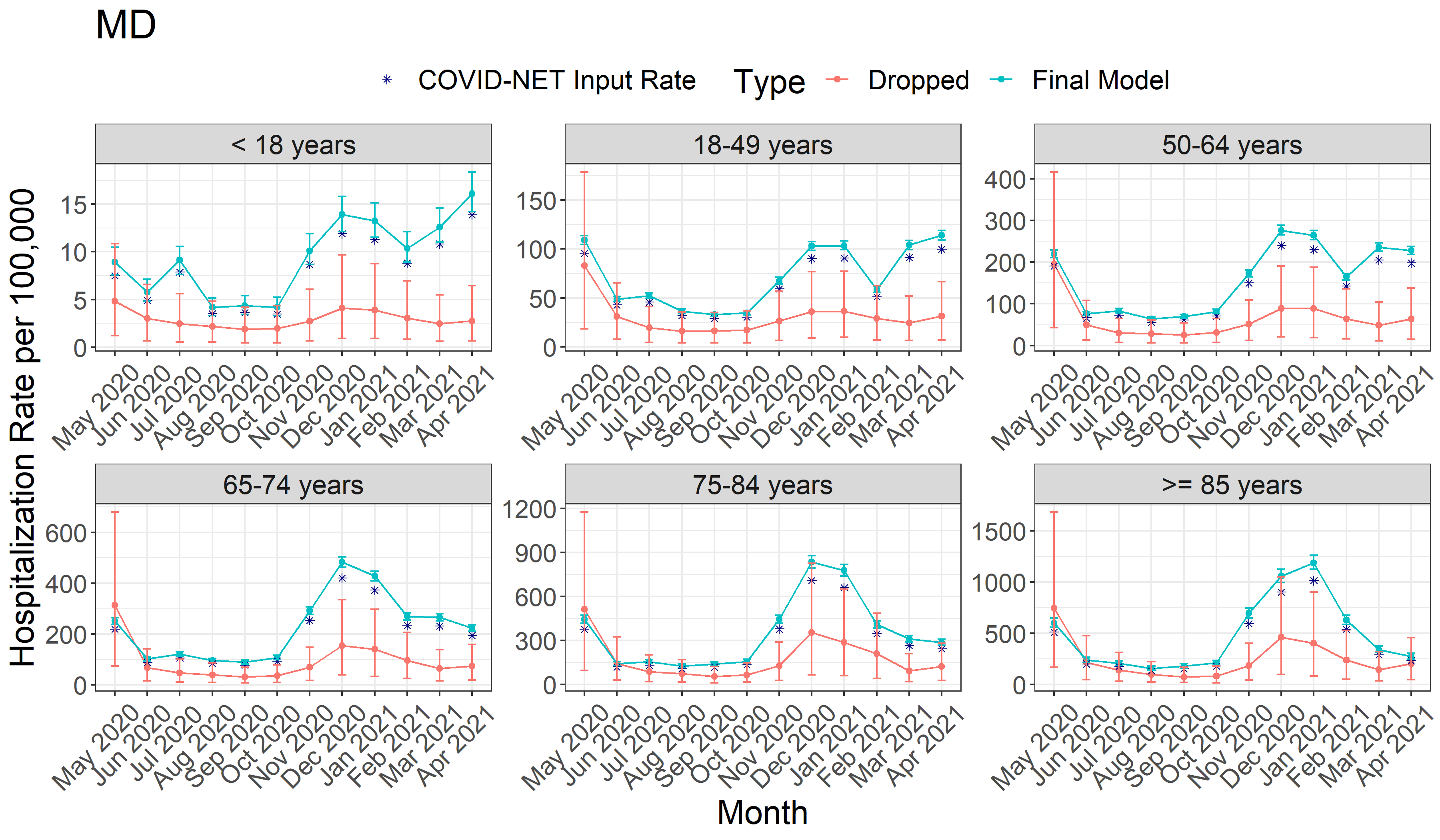


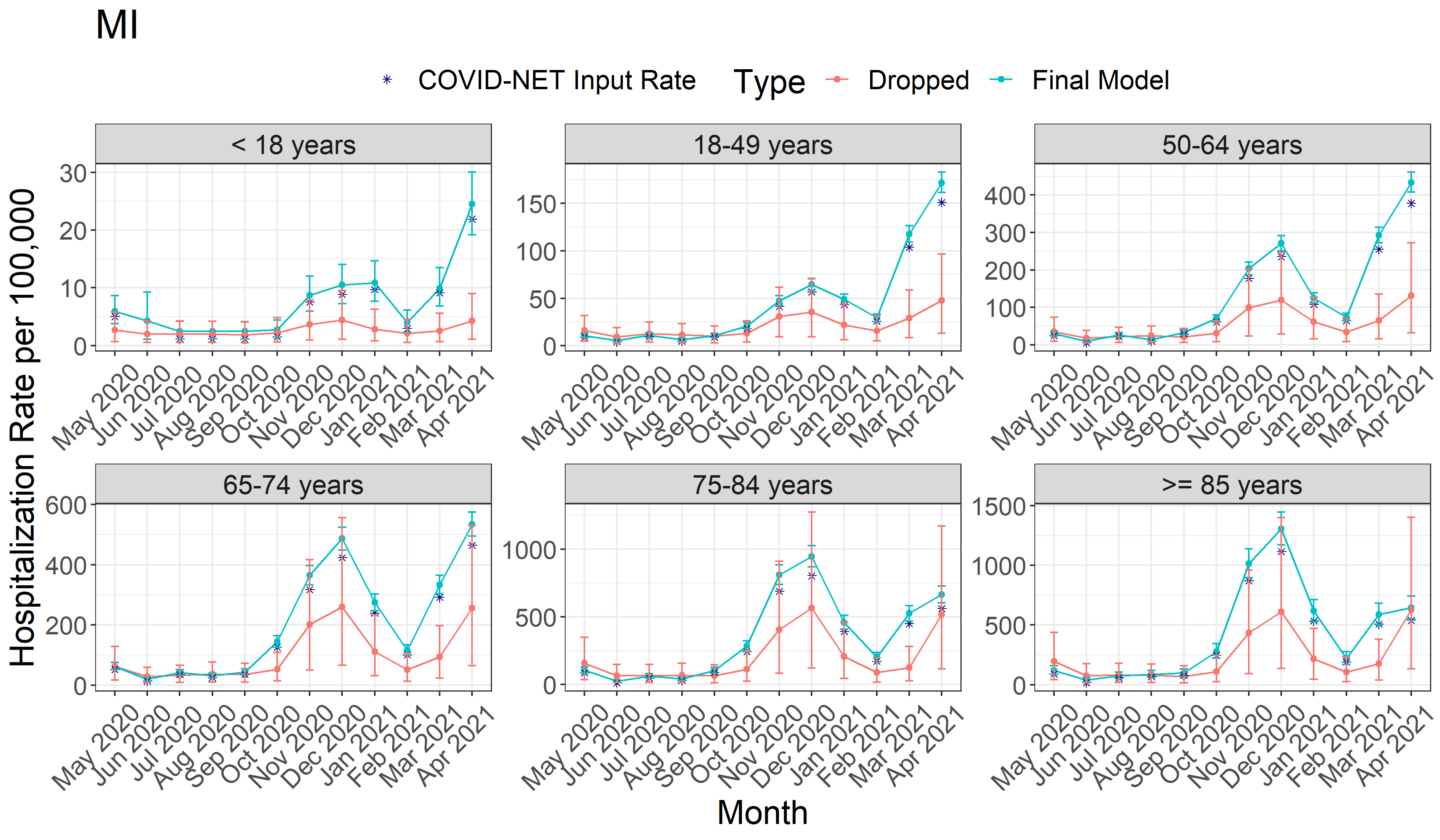


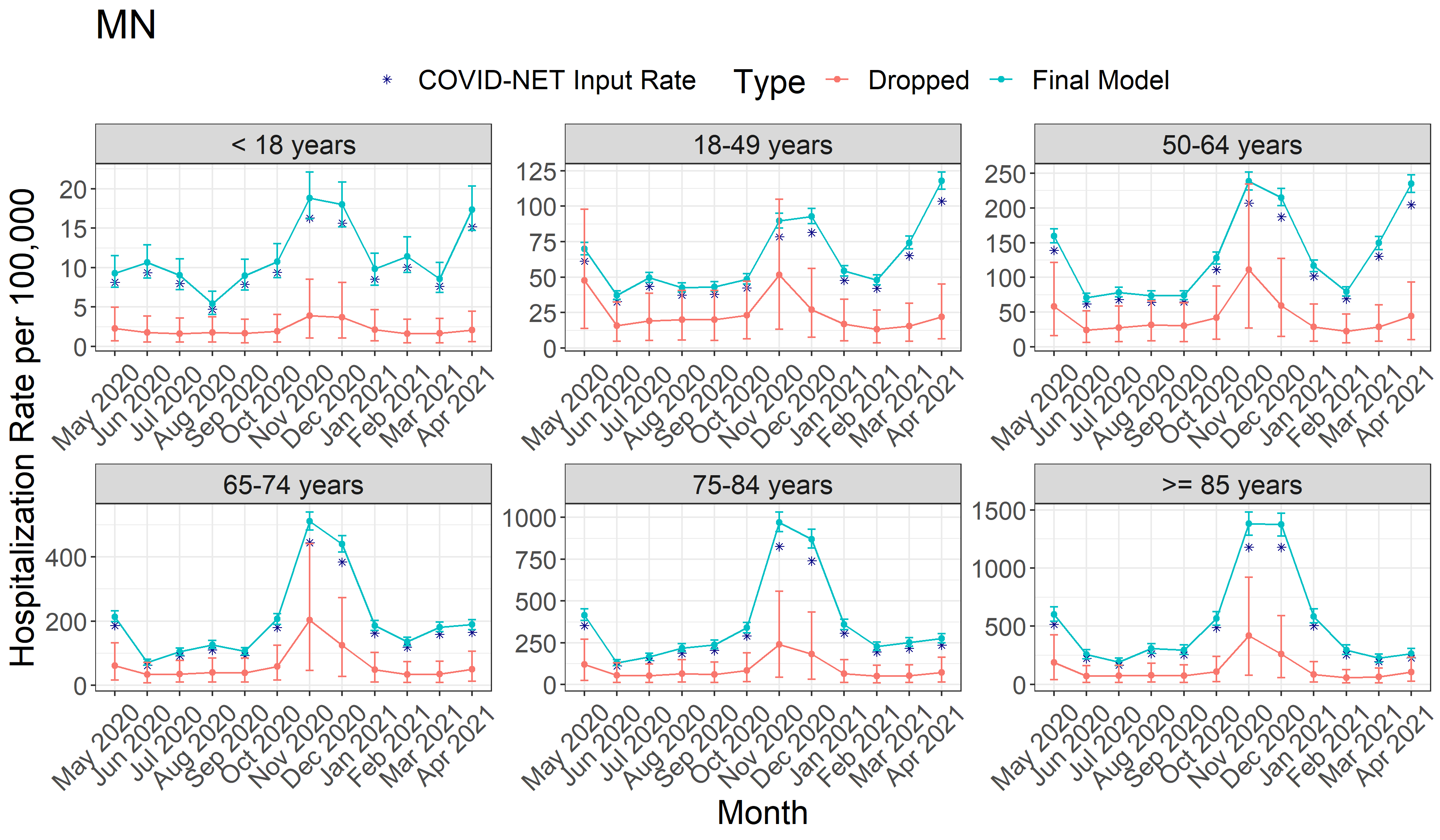


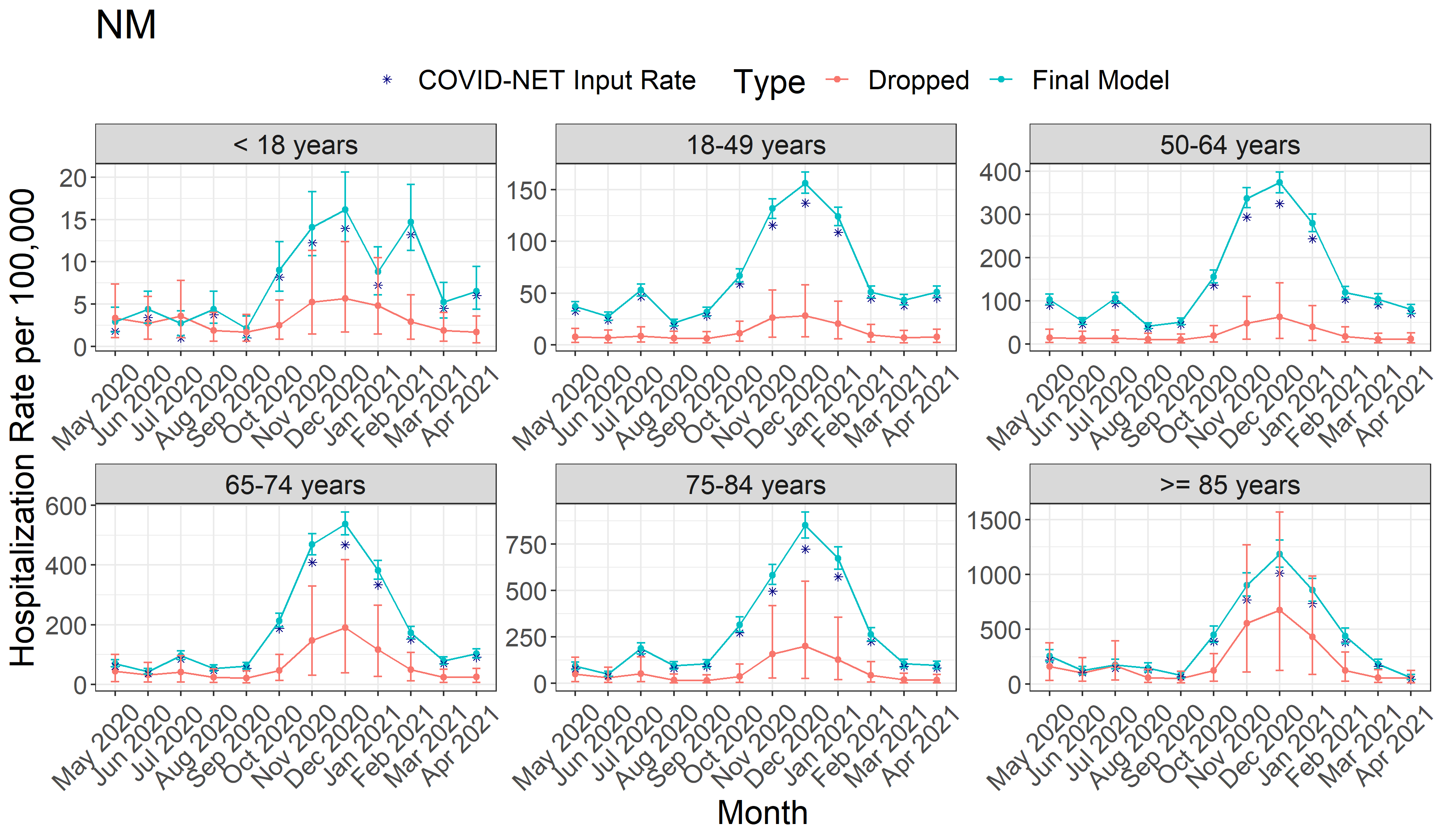


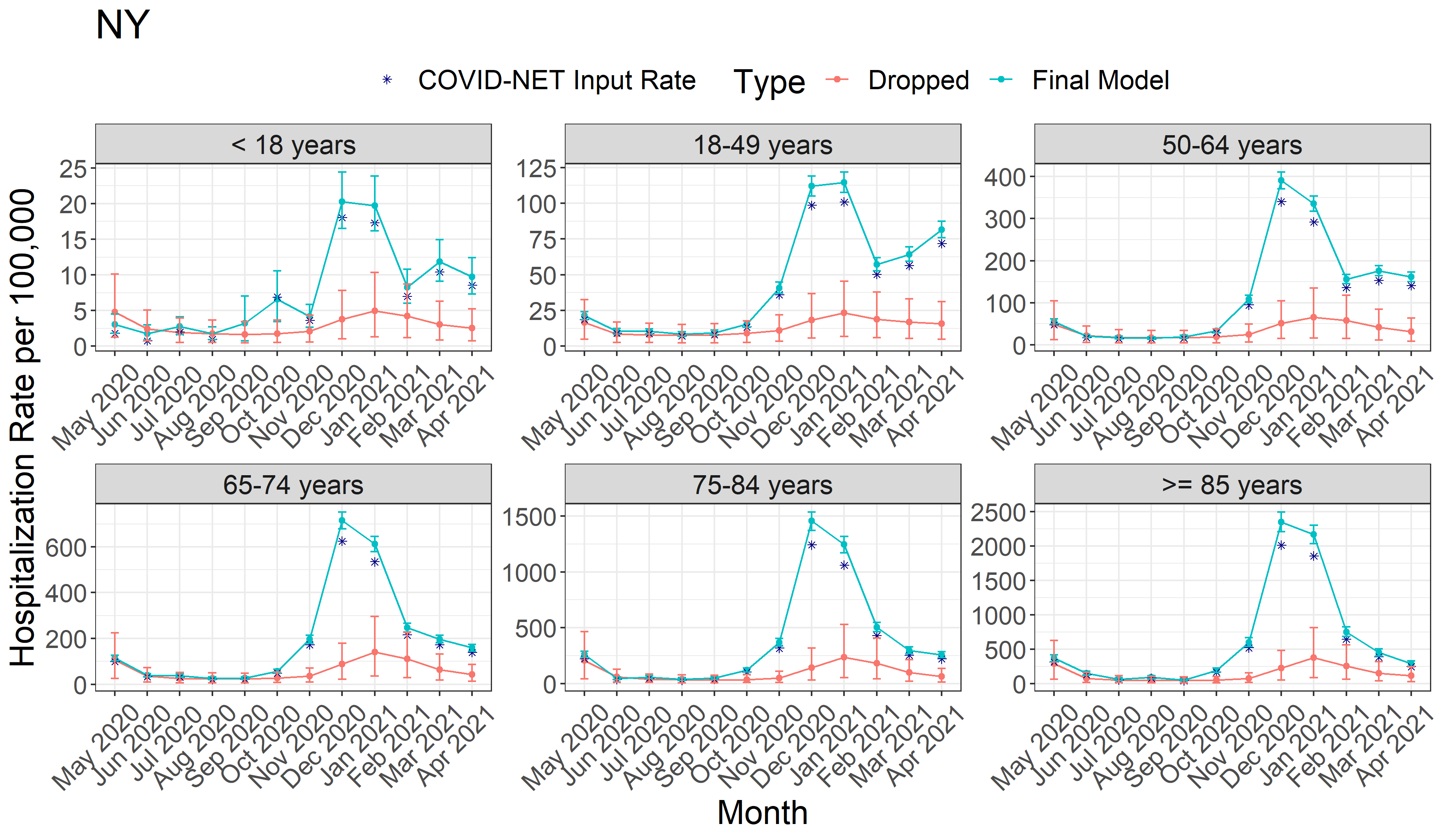


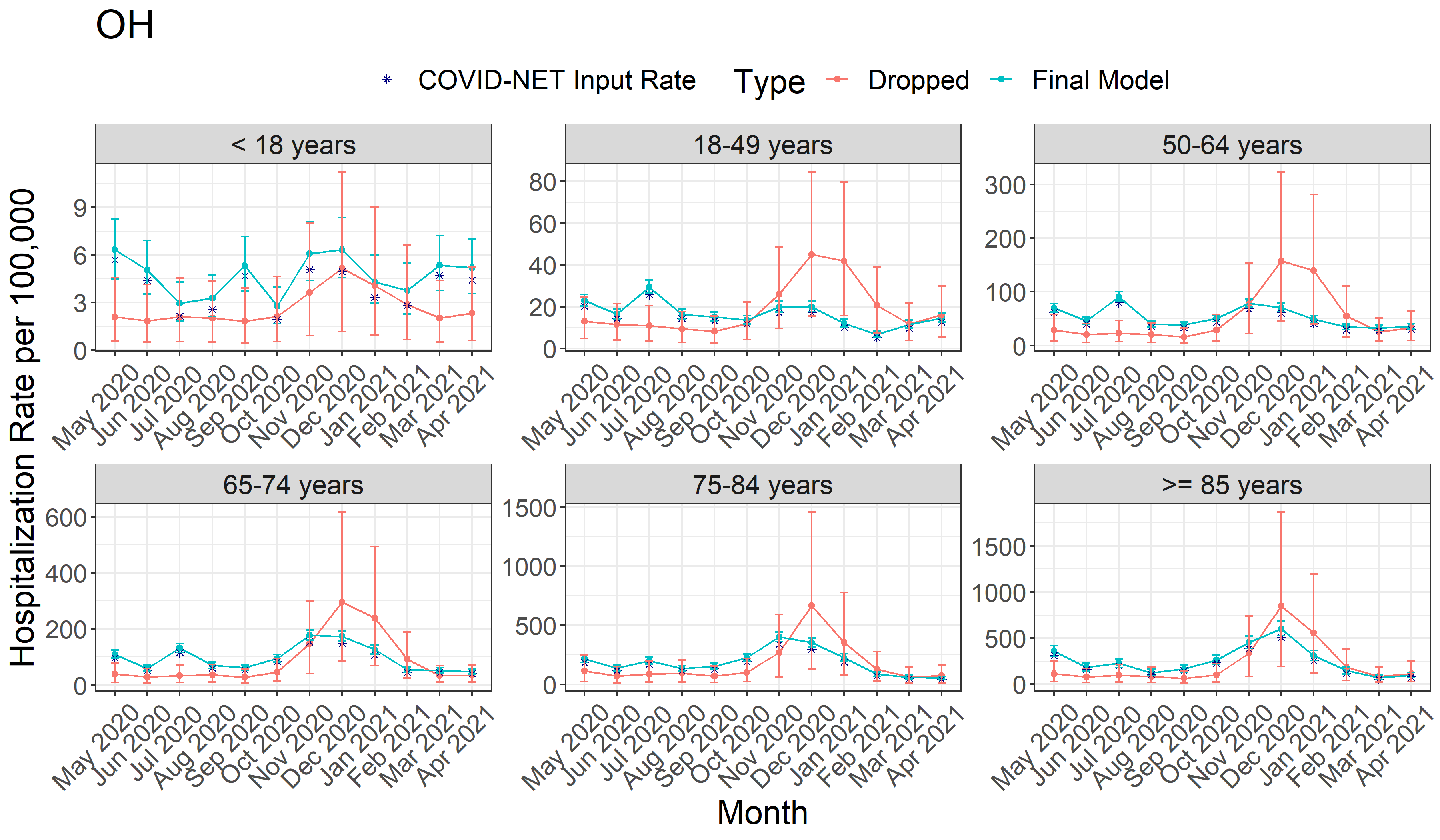


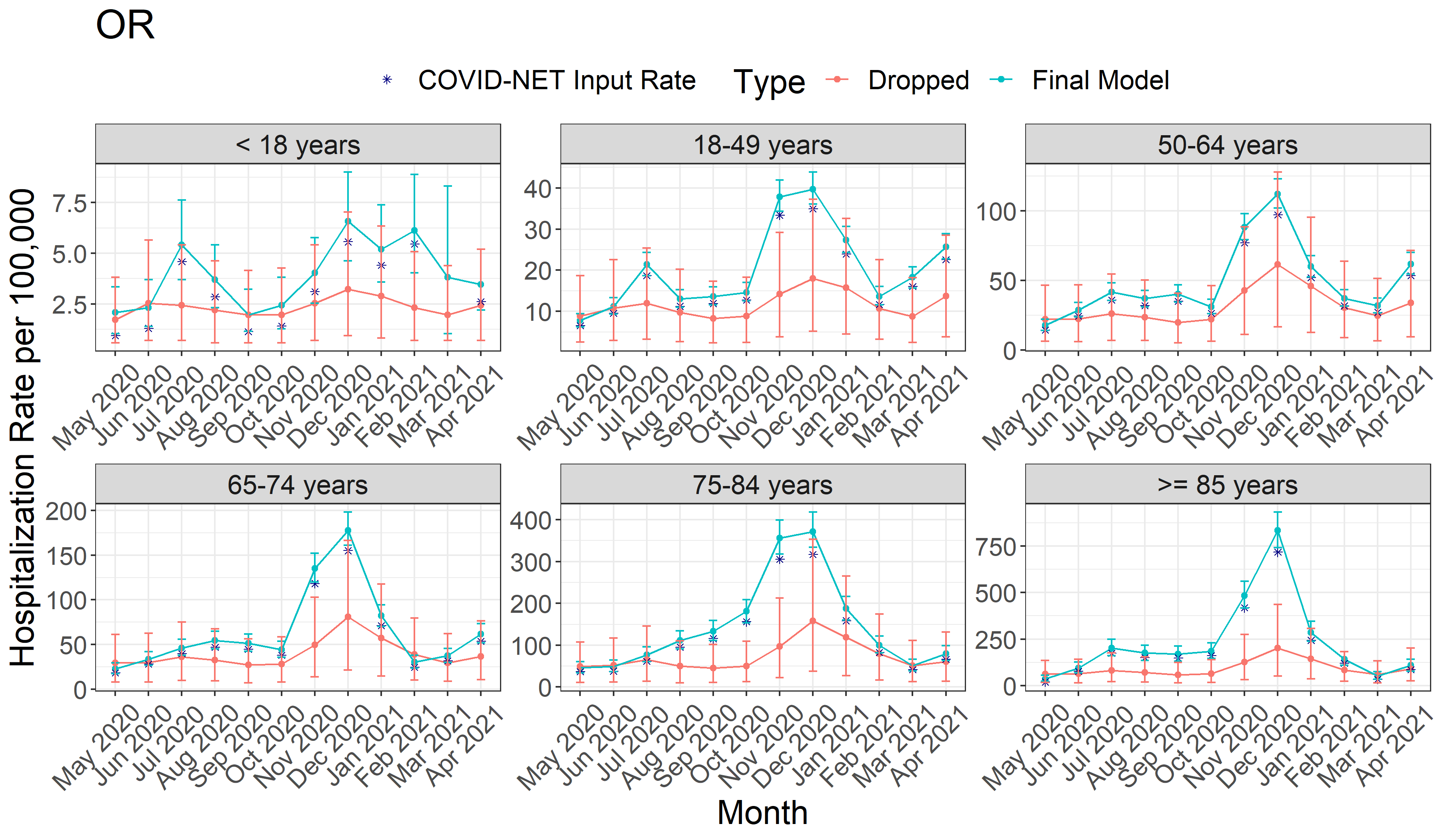


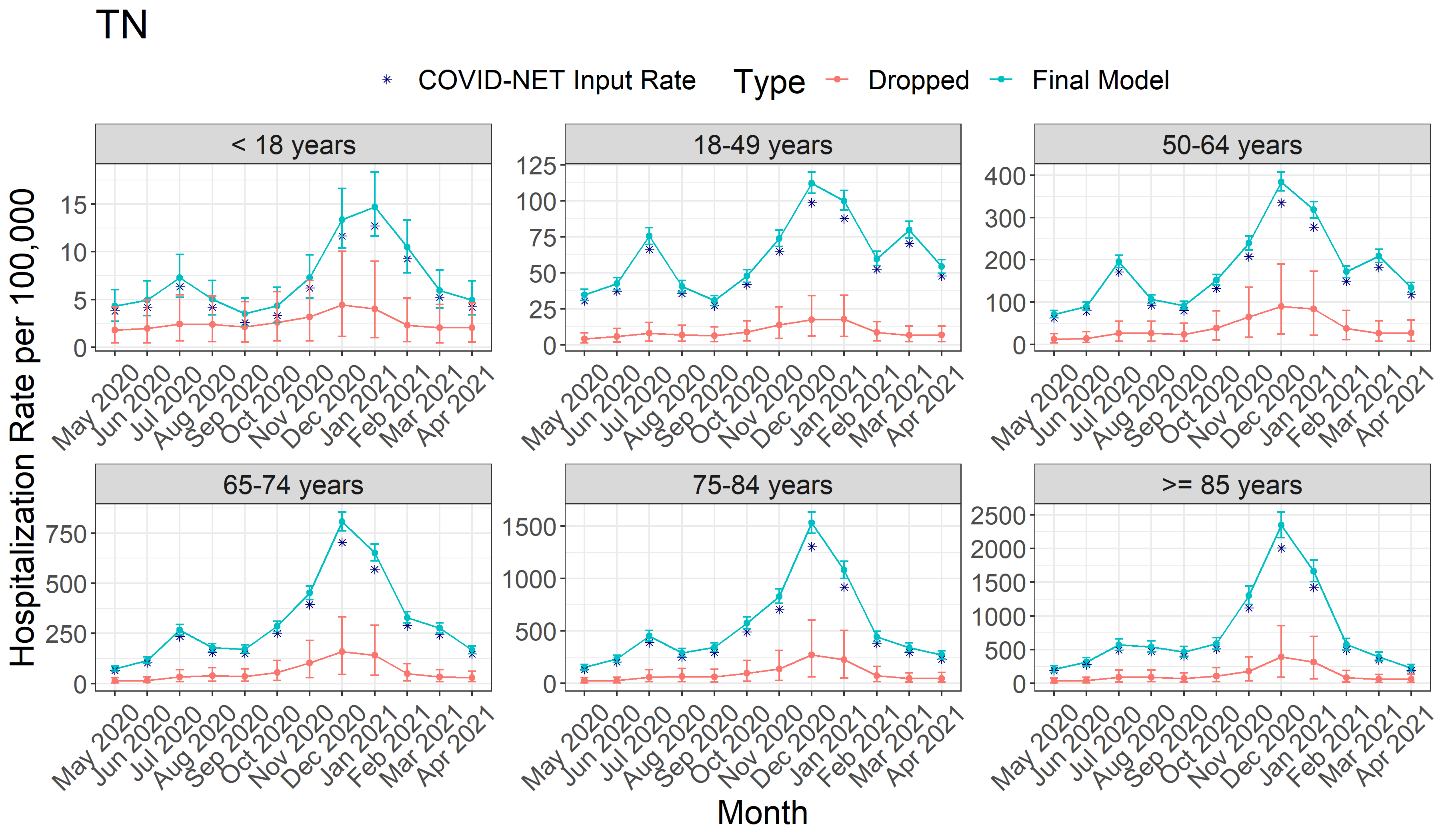


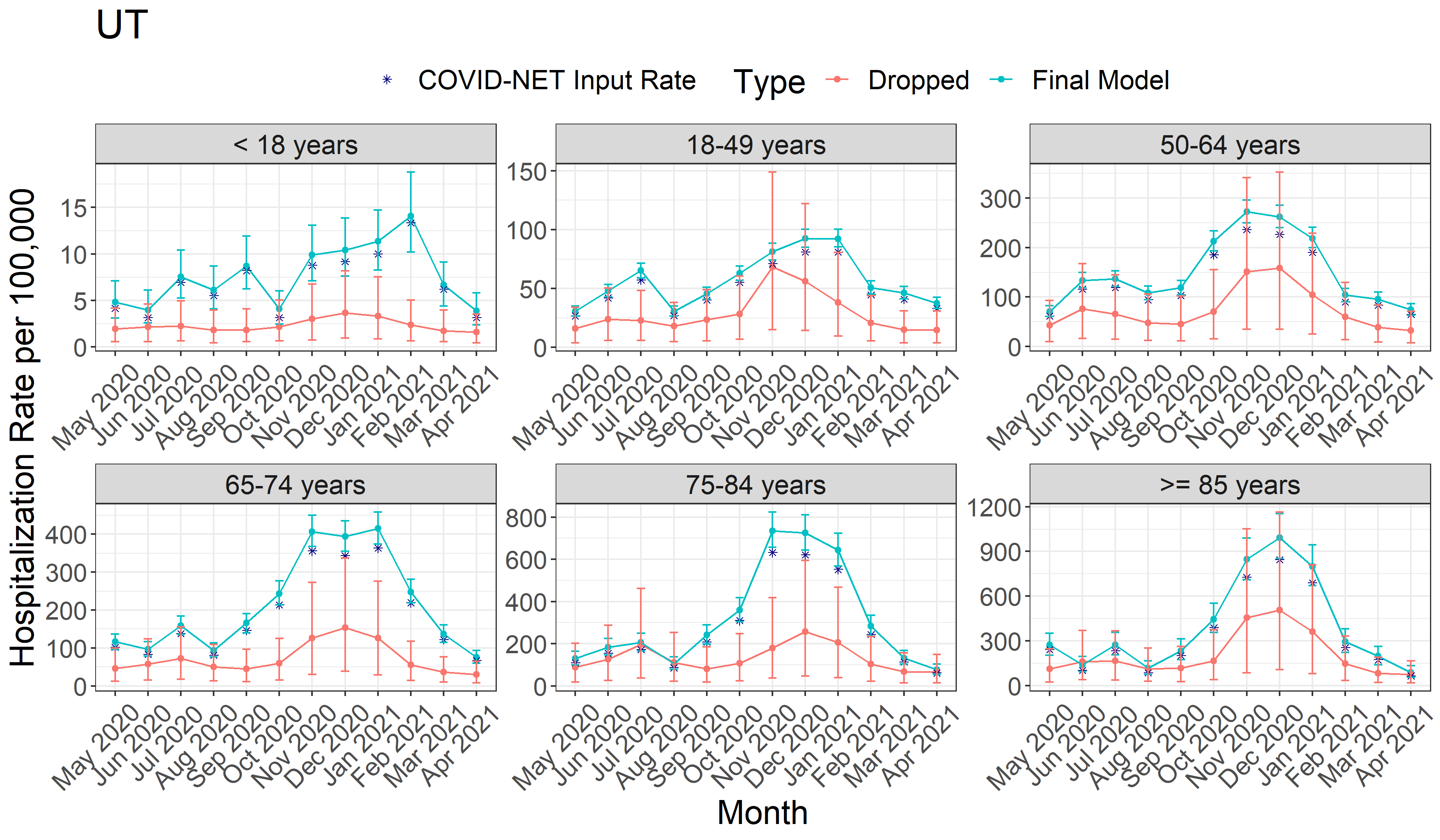

Supplement: Multimedia Appendix 2 [file publichealth_v8i6e34296_app2.docx]
