# Supplementary material for: Estimating COVID-19 Hospitalizations in the United States With Surveillance Data Using a Bayesian Hierarchical Model: Modeling Study
Source: JMIR Public Health Surveill. 2022 Jun 2;8(6):e34296. doi: 10.2196/34296 (PMC9169704; doi:10.2196/34296)
Supplement: Multimedia Appendix 3 [file publichealth_v8i6e34296_app3.docx]

## Multimedia Appendix 3

Table S2: Comparison of COVID-19 hospitalization estimates between our Bayesian model and case-based multiplier model by age group, months, and HHS regions, including distribution of hospitalization for each group from June 2020 through March 2021.

| **Group** | **Final Bayesian Model** | | **Case-Based Multiplier Model** | |
| --- | --- | --- | --- | --- |
|  | **Hospitalization Count (% of Group)** | **Hospitalization Rate per 100,000** | **Hospitalization Count (% of Group)** | **Hospitalization Rate per 100,000** |
| **Age Groups** |  |  |  |  |
| Ages 0-17 | 53,400 (1.6) | 67.4 | 167,085 (3.6) | 210.7 |
| Ages 18-49 | 779,800 (24.9) | 519.1 | 1,124,575 (24.1) | 748.6 |
| Ages 50-64 | 786,900 (25.1) | 1252.6 | 1,185,968 (25.5) | 1887.9 |
| Ages 65+ | 1,511,600 (48.3) | 2800.8 | 2,181,343 (46.8) | 4041.7 |
| **Months** |  |  |  |  |
| June - July | 461,300 (14.7) | 138.9 | 572,148 (12.3) | 172.4 |
| August - September | 362,600 (11.6) | 110.7 | 404,311 (8.7) | 123.4 |
| October - November | 606,700 (19.4) | 185.2 | 1,008,710 (21.7) | 308.0 |
| December - January | 1,207,500 (38.6) | 368.7 | 1,585,383 (34.0) | 484.0 |
| February - March | 493,600 (15.8) | 147.4 | 1,088,419 (23.4) | 325.0 |
| **HHS Regions** |  |  |  |  |
| Reg1 | 171,000 (5.5) | 1152.2 | 148,947 (3.2) | 1003.3 |
| Reg2 | 262,200 (8.4) | 925.5 | 797,480 (17.1) | 2814.4 |
| Reg3 | 260,000 (8.3) | 692.2 | 327,607 (7.0) | 872.2 |
| Reg4 | 682,000 (21.8) | 1019.3 | 625,422 (13.4) | 934.7 |
| Reg5 | 423,900 (13.5) | 806.7 | 815,382 (17.5) | 1551.9 |
| Reg6 | 577,800 (18.4) | 1352.6 | 672,674 (14.4) | 1574.7 |
| Reg7 | 171,200 (5.5) | 1210.4 | 148,695 (3.2) | 1051.6 |
| Reg8 | 103,300 (3.3) | 618.8 | 104,509 (2.2) | 626.1 |
| Reg9 | 404,500 (12.9) | 788.6 | 940,347 (20.2) | 1833.5 |
| Reg10 | 75,800 (2.4) | 528.3 | 77,908 (1.7) | 542.9 |
| **Total** | 3,119,400 | 904.3 | 4,658,971 | 1345..3 |
